# Comparison of crystal structure and DFT calculations of triferrocenyl trithiophosphite’s conformance

**DOI:** 10.3762/bjoc.18.157

**Published:** 2022-10-25

**Authors:** Ruslan P Shekurov, Mikhail N Khrizanforov, Ilya A Bezkishko, Tatiana P Gerasimova, Almaz A Zagidullin, Daut R Islamov, Vasili A Miluykov

**Affiliations:** 1 Arbuzov Institute of Organic and Physical Chemistry, FRC Kazan Scientific Center, Russian Academy of Sciences; 2 Aleksander Butlerov Institute of Chemistry, Kazan Federal University, Kazan, 420008, 1/29 Lobachevskogo str., Russian Federation; 3 Laboratory for Structural Studies of Biomacromolecules, FRC Kazan Scientific Center of RAS, Lobachevskogo Street 2/31, Kazan 420111, Russian Federation

**Keywords:** DFT calculations, multi-ferrocenyl compounds, phosphorus thioesters, trithiophosphite, X-ray

## Abstract

A triferrocenyl trithiophosphite was studied by X-ray single-crystal diffraction. Triferrocenyl trithiophosphite has nine axes of internal rotation: three P–S bonds, three C–S bonds and three Fe–cyclopentadienyl axes. Rotation around the P–S bonds results in a totally asymmetric structure with three ferrocenylthio groups exhibiting different orientations towards the phosphorus lone electron pair (LEP). A comparison of DFT calculations and X-ray diffraction data is presented, herein we show which conformations are preferred for a given ligand.

## Introduction

The design of novel “stimuli-responsive” molecules is a very attractive area in modern chemistry due to a number of various practical applications of such compounds [1–6]. Multiferrocenes are of particular fundamental interest because of their multistep electrochemical and magnetic properties. Such switchable systems with conjugated organic fragments containing an Fe^II^/Fe^III^ system were used in organic electronics as molecular switches, optoelectronic materials and in biochemistry as photonic or redox devices [6].

A promising approach is the coordination self-assembly of multiferrocene ensembles from ferrocene-containing ligands and metal ions or clusters. This makes it possible to realize an almost infinite number of multiferrocene compounds and to select leading compounds for the successful creation of molecular electronic devices. It should be noted that with the exception of tertiary phosphines, a relatively small number of trivalent phosphorus derivatives has been used to construct multiferrocene compounds. The use of ferrocene derivatives containing a phosphorus–sulfur bond is a promising direction, since coordination with a metal atom can occur both at the phosphorus and sulfur atoms [7]. It is important to know the conformational capabilities of such ligands for construction of such complexes [8–11].

However, to date, XRD data on phosphorus derivatives containing a ferrocenyl substituent at the sulfur atom are presented only in oxidized and sulfurized forms. Trithiophosphite has not been studied by X-ray diffraction analysis, although it is of great interest for the construction of complexes with multiferrocene systems. Herein we present for the first time X-ray diffraction data of (FcS)_3_P and compare it with DFT calculations to show which conformation are preferred for a given ligand.

## Experimental

### General

All reactions and manipulations were carried out under dry pure N_2_ using standard Schlenk techniques. All solvents were distilled from sodium/benzophenone and stored under nitrogen before use. The NMR spectra were recorded on a Bruker MSL-400 spectrometer (^1^H 400 MHz, ^31^P 161.7 MHz, ^13^C 100.6 MHz). SiMe_4_ was used as internal reference for ^1^H NMR chemical shifts, and 85% H_3_PO_4_ as external reference for ^31^P NMR. The elemental analyses were carried out at the microanalysis laboratory of the Arbuzov Institute of Organic and Physical Chemistry, Russian Academy of Sciences.

### Synthesis

To a suspension of white phosphorus (0.08 g, 0.645 mmol) in acetone (30 mL) were added diferrocenyldisulfide (1.68 g, 3.8 mmol) and 0.2 mL 15 N solution of potassium hydroxide. The reaction mixture was stirred for 12 h at room temperature and then the solvent was evaporated in vacuo. The product was extracted with benzene (3 × 30 mL) and after evaporation of the solvent triferrocenyl trithiophosphite (1.34 g, 76%) was obtained as a yellow powder. Single crystals suitable for X-ray diffraction were obtained by dissolving the compound in a mixture of benzene/hexane 1:1 and storing the solution in a fridge.

Mp 200–203 °C; ^1^H NMR (400 MHz, C_6_D_6_, δ) 4.56 (m, 6 H_β_), 4.03 (m, 6 H_α_), 4.14 (s, 15H); ^31^P NMR (161.7 MHz, C_6_D_6_, δ) 126.6; Anal. calcd for C_30_H_27_Fe_3_PS_3_ (760.37): C, 52.82; H, 3.99; P, 4.54; S, 14.09; found: C, 52.84; H, 3.96; P, 4.49; S, 14.04.

### Single crystal X-ray diffraction

The data set for single crystals of triferrocenyl trithiophosphite was collected on a Rigaku XtaLab Synergy S instrument with a HyPix detector and a PhotonJet microfocus X-ray tube using Cu Kα (1.54184 Å) radiation at 100 K. Images were indexed and integrated using the CrysAlisPro data reduction package. The data were corrected for systematic errors and absorption using the ABSPACK module. The GRAL module was used for analysis of systematic absences and space group determination. Using Olex2 [11], the structure was solved by direct methods with SHELXT [12] and refined by the full-matrix least-squares on F^2^ using SHELXL [13]. Non-hydrogen atoms were refined anisotropically. The figures were generated using the Mercury 4.1 program [14].

**Crystal data** for C_30_H_27_Fe_3_PS_3_ (*M* = 682.21 g/mol): monoclinic, space group *P*2_1_/*c* (no. 14), *a* = 7.49490(10) Å, *b* = 19.8932(3) Å, *c* = 18.4291(3) Å, β = 99.792(2)°, *V* = 2707.70(7) Å^3^, *Z* = 4, *T* = 100.0(5) K, μ(Cu Kα) = 15.586 mm^−1^, *D*_calc_ = 1.674 g/cm^3^, 17211 reflections measured (6.59° ≤ 2Θ ≤ 153.132°), 5496 unique (*R*_int_ = 0.0570, *R*_sigma_ = 0.0467) which were used in all calculations. The final *R*_1_ was 0.0496 (I > 2σ(I)) and *wR*_2_ was 0.1349 (all data). CCDC number 2201898.

### DFT calculations

All calculations were performed with the Gaussian 16 suite of programs [15]. The hybrid PBE0 functional [16] and the Ahlrichs’ triple-ζ def-TZVP AO basis set [17] were used for optimization of all structures. In all geometry optimizations, the D3 approach [18] was applied to describe the London dispersion interactions as implemented in the Gaussian 16 program.

## Results and Discussion

Previous electrochemical studies for triferrocenyl trithiophosphite revealed in their cyclovoltammograms three reversible one-electron peaks corresponding to stepwise oxidation of the three ferrocene moieties. It should be noted that the first oxidation potential is almost identical to free ferrocene [6]. Herein we report the crystal structure of triferrocenyl trithiophosphite.

For triferrocenyl trithiophosphite a *trans*-*gauche*-*gauche* configuration with torsion angles of −34°, −40°, and 173°, respectively, has been observed, although a propeller-like *gauche*-*gauche*-*gauche* configuration of alkyl(aryl)thio groups has been observed for trithiophosphites even in the solid state [7] or in the gas phase [8–10].

**Figure 1:**
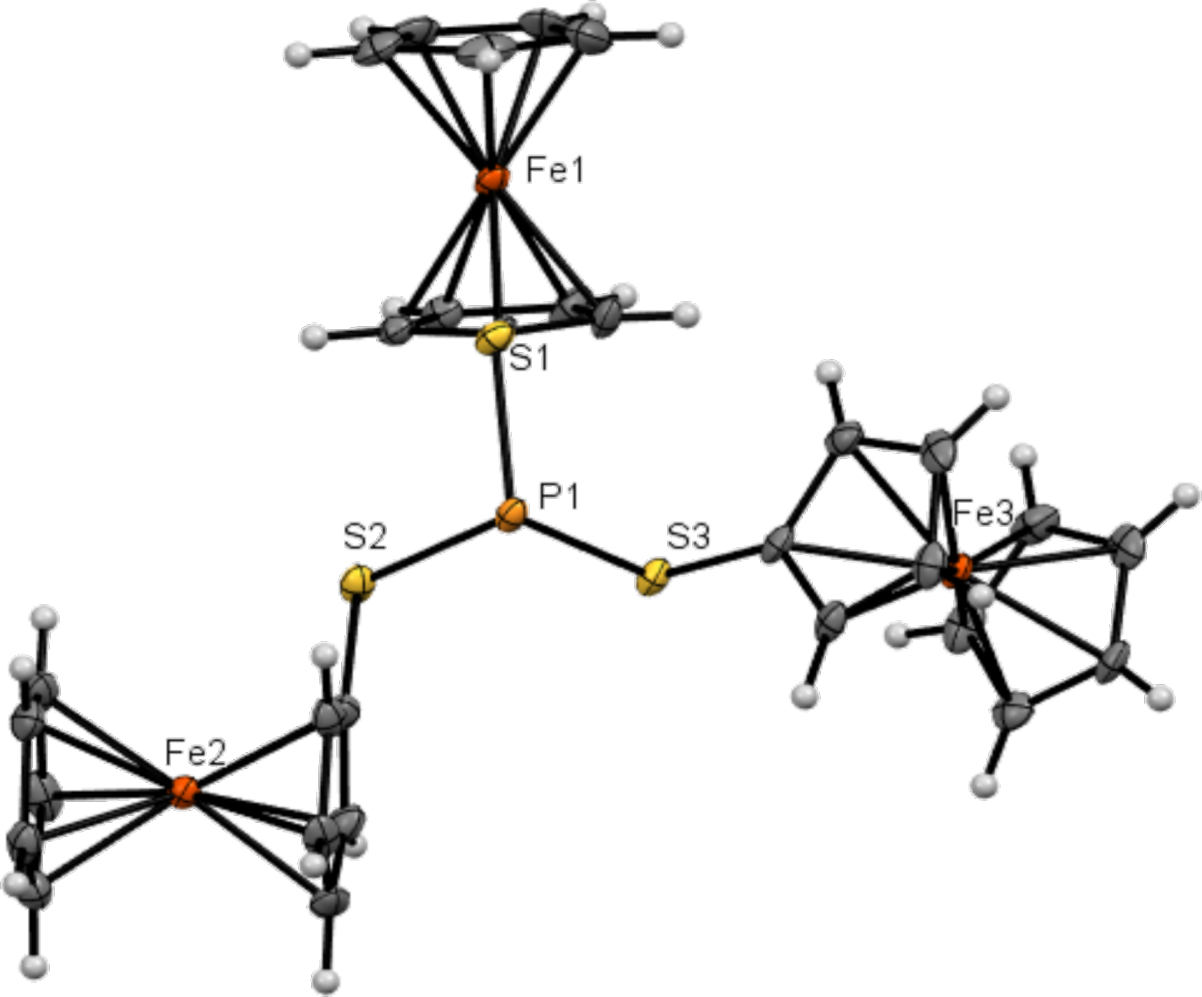
ORTEP representation of triferrocenyl trithiophosphite showing 50% probability thermal ellipsoids.

Triferrocenyl trithiophosphite has nine axes of internal rotation: three P–S bonds, three C–S bonds, and three Fe–cyclopentadienyl axes. The rotation around the P–S bonds results in a totally unsymmetrical structure with three ferrocenylthio groups exhibiting different orientations towards the phosphorus lone electron pair (Figure 1).

**Figure 2:**
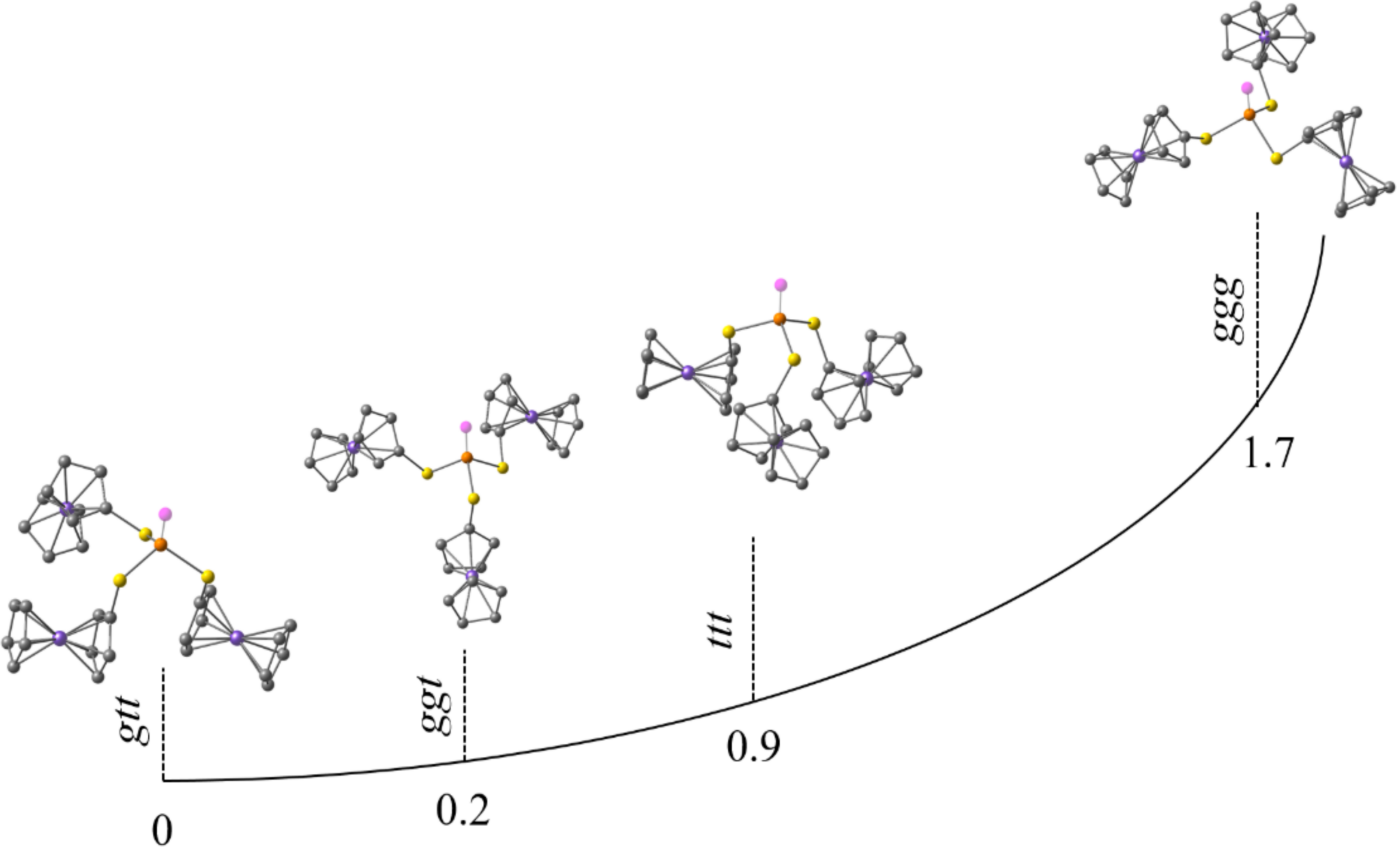
Optimized conformations and relative energies of four possible conformers of triferrocenyl trithiophosphite.

Several possible conformations of triferrocenyl trithiophosphite have been considered quantum-chemically (Figure 2, Table 1): *trans*-*trans*-*trans* (*ttt*), *gauche*-*trans*-*trans* (*gtt*), *gauche*-*gauche*-*trans* (*ggt*), and *gauche*-*gauche*-*gauche* (*ggg*). During optimization the *ggt* conformer adopted a *cis*-*gauche*-*trans* conformation with Fc(C)–S–P lone pair dihedral angles of 8°, −60°, and 173°, respectively (Table 1). The lowest energy has been predicted for the *gtt* conformer, nevertheless the energy differences between the *gtt* and *cgt* conformers are negligible (0.23 kcal/mol). Interestingly, the *cgt* conformation has been found previously for tricymantrenyl trithiophosphite [19]. The highest relative energy is predicted for the *ggg* conformer (1.7 kcal/mol). The ferrocene adopts an almost eclipsed conformation in all the models with the dihedral angle between two Cp rings of ≈ 10°. Our previous work indicated that Cp can rotate at room temperature [20]. The Fc(C)–S–P lone pair dihedral angle for the *ttt* conformer is ≈ 150°, and for the *ggg* conformer it is ≈ −35°. For the *gtt*/*cgt* conformers the *trans* S–Fc bonds are almost antiparallel to the phosphorus lone pair (LEP): 175°, −161°/173°. The dihedral angle for the *gauche* S–Fc bond in the *gtt* conformer is −56°, and a close value is predicted for one of the *gauche* S–Fc bonds in the *tgg* conformer (−60°), whereas the second one is almost parallel to LEP (8°).

**Table 1:** Calculated relative energies and dihedral angles Fc(C)–S–P=X (°) (X = LEP, O, S) of four possible conformers of (FcS)_3_P, (FcS)_3_PO, and (FcS)_3_PS.

	(FcS)_3_P	(FcS)_3_PO	(FcS)_3_PS

*ttt*	0.91	**0**	0.04
	149/151/151	149/149/149	149/149/149
*gtt*	**0**	0.23	0.20
	−56/175/−161	−56/−173/−135	47/174/135
*ggt/cgt*	0.23	0.52	0.36
	8/−60/173	−62/−47/165	46/45/176
*ggg*	1.73	0.55	**0**
	−37/−35/−36	−52/−34/−53	42/44/44

**Figure 3:**
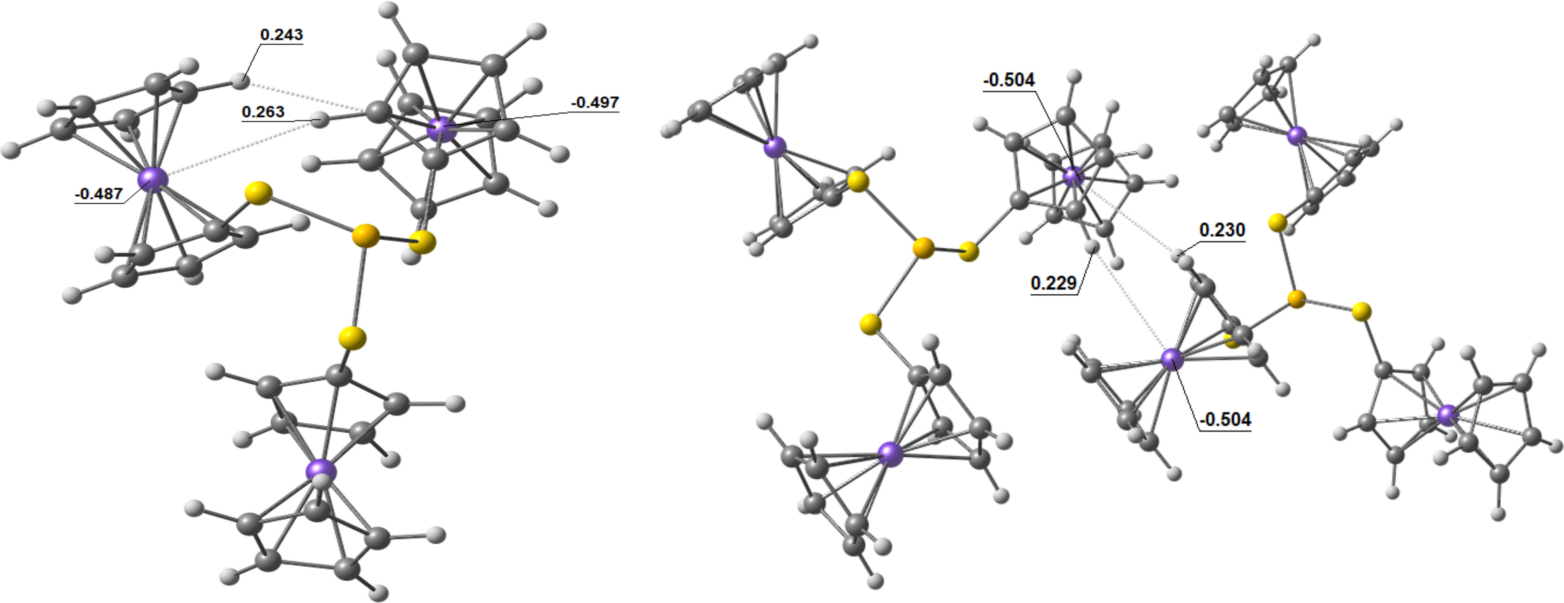
Calculated NBO charges on the Fe ions and hydrogen atoms for the optimized *ttg* conformer (left) and for two neighboring molecules (right) from X-ray analysis data.

The energy difference between the considered conformations is quite small, suggesting other factors playing a significant role. The highest energy predicted for the *ggg* conformer is obviously related to the absence of stabilizing intramolecular CH···π (like in the *gtt* and *cgt* cases) or CH···Fe (like in the *ttt* case) interactions between neighboring fragments in the structure. The latter plays an important role from the electrostatic point of view; the NBO analysis predict a negative charge at the Fe ion and positive charges at hydrogen atoms (Figure 3). Thus the crystal structure of (FcS)_3_P is defined rather by plural intermolecular interactions than by relative energetics of conformers (Figures S1–S3 in Supporting Information File 1).

**Figure 4:**
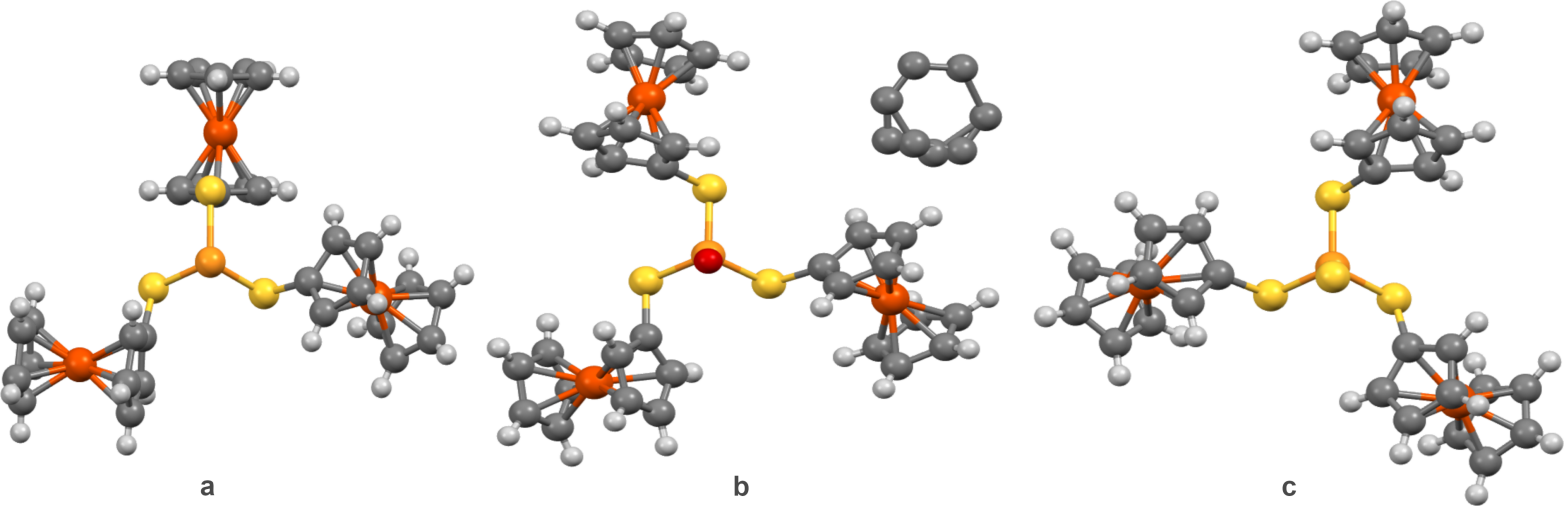
Molecular structures in the solid state of a) (FcS)_3_P, b) (FcS)_3_PO [19], and c) (FcS)_3_PS [7] as established by single crystal X-ray diffraction analyses. C atoms – grey, Fe atoms – brown, O atoms – red, P atoms – orange, S atoms – yellow.

Previously, for triferrocenyl trithiophosphate and triferrocenyl tetrathiophosphate with P=O and P=S moieties propeller-like *ggg* conformations have been found by X-ray diffraction analysis. Indeed, computations predict the *ggg* conformer to be the most energetically advantageous for the P=S containing compound, however with very close energies of the *ttt* and the *ggg* conformers (Table 1). For the P=O containing compound the *ttt* conformer is predicted to have the lowest energy. Nevertheless for both P=X compounds computations predict very small energy differences between all four conformers, lower than 0.6 kcal/mol. Thus, one can conclude that in these cases crystal packing influences the conformation. A comparison of the crystal packings for the P_LEP_, P=O, and P=S containing compounds clearly confirms this conclusion experimentally (Figure 4).

We compared the crystal packings of three similar compounds: (FcS)_3_P, (FcS)_3_PO [19], and (FcS)_3_PS [7] (Figure 4). All three compounds form crystals belonging to the monoclinic syngony. In all three cases, the molecules in the crystals form a herringbone motif. In (FcS)_3_P, C–H···π interactions dominate, while in (FcS)_3_PS and (FcS)_3_PO, in addition to C–H···π interactions, by one C–H···S and two C–H···O interactions, respectively, are observed. It should be noted that (FcS)_3_PO crystals contain a solvent molecule that participates in intermolecular interactions. Thus, despite the similarity of the molecular structure of the three compounds and some crystal parameters, the intermolecular interactions differ noticeably from each other.

**Figure 5:**
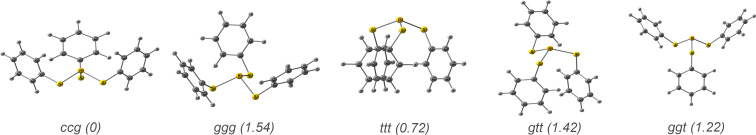
Quantum chemically optimized conformations of the (PhS)_3_P molecule and their relative energies (kcal/mol).

At the same time one should underline the role of the ferrocene moiety for the crystal structure of the (FcS)_3_P. The related (PhS)_3_P molecule with Ph rings instead of Fc units exist in the propeller-like *gauche*-*gauche*-*gauche* configuration [21], forming the C–H···π-bonded dimers (Figures S4 and S5 in Supporting Information File 1). The computations of the relative energies of five possible conformers of (PhS)_3_P (*ggg*, *ttt*, *ttg*, *ggt*, *ccg*) predict the lowest energy for the *ccg* conformation (Figure 5). The propeller-like *ggg* conformer found in the solid state has the highest energy. Most obviously the latter is stabilized by intermolecular C–H···π interactions (Figures S4 and S5 in Supporting Information File 1). The bulky Fc moieties do not allow to form such type of dimers.

## Conclusion

Triferrocenyl trithiophosphite (FcS)_3_P was studied by X-ray single-crystal diffraction for the first time. DFT calculations and X-ray diffraction data were compared, and the preferred conformations were established. Despite the similarity of the molecular structures and some crystal parameters of (FcS)_3_P, (FcS)_3_PO, and (FcS)_3_PS, the intermolecular interactions differ noticeably from each other.

## Supporting Information

File 1 Additional figures.

File 2 CIF file for triferrocenyl trithiophosphite.

File 3 Check-CIF file for triferrocenyl trithiophosphite.
