# Supplementary material for: Comparison of crystal structure and DFT calculations of triferrocenyl trithiophosphite’s conformance
Source: Beilstein J Org Chem. 2022 Oct 25;18:1499–504. doi: 10.3762/bjoc.18.157 (PMC9643052; doi:10.3762/bjoc.18.157)
Supplement: File 1 — Additional figures. [file Beilstein_J_Org_Chem-18-1499-s001.pdf]

BEILSTEIN JOURNAL OF ORGANIC CHEMISTRY

Supporting Information

for

Comparison of crystal structure and DFT calculations of triferrocenyl trithiophosphite's conformance

Ruslan P. Shekurov, Mikhail N. Khrizanforov, Ilya A. Bezkishko, Tatiana P. Gerasimova, Almaz A. Zagidullin, Daut R. Islamov and Vasili A. Miluykov

Beilstein J. Org. Chem. **2022,** *18,* 1499–1504. doi:10.3762/bjoc.18.157

Additional figures

License and Terms: This is a supporting information file under the terms of the Creative Commons Attribution License (https://creativecommons.org/licenses/by/4.0). Please note that the reuse, redistribution and reproduction in particular requires that the author(s) and source are credited and that individual graphics may be subject to special legal provisions.
The license is subject to the *Beilstein Journal of Organic Chemistry* terms and conditions: (https://www.beilstein-journals.org/bjoc/terms)

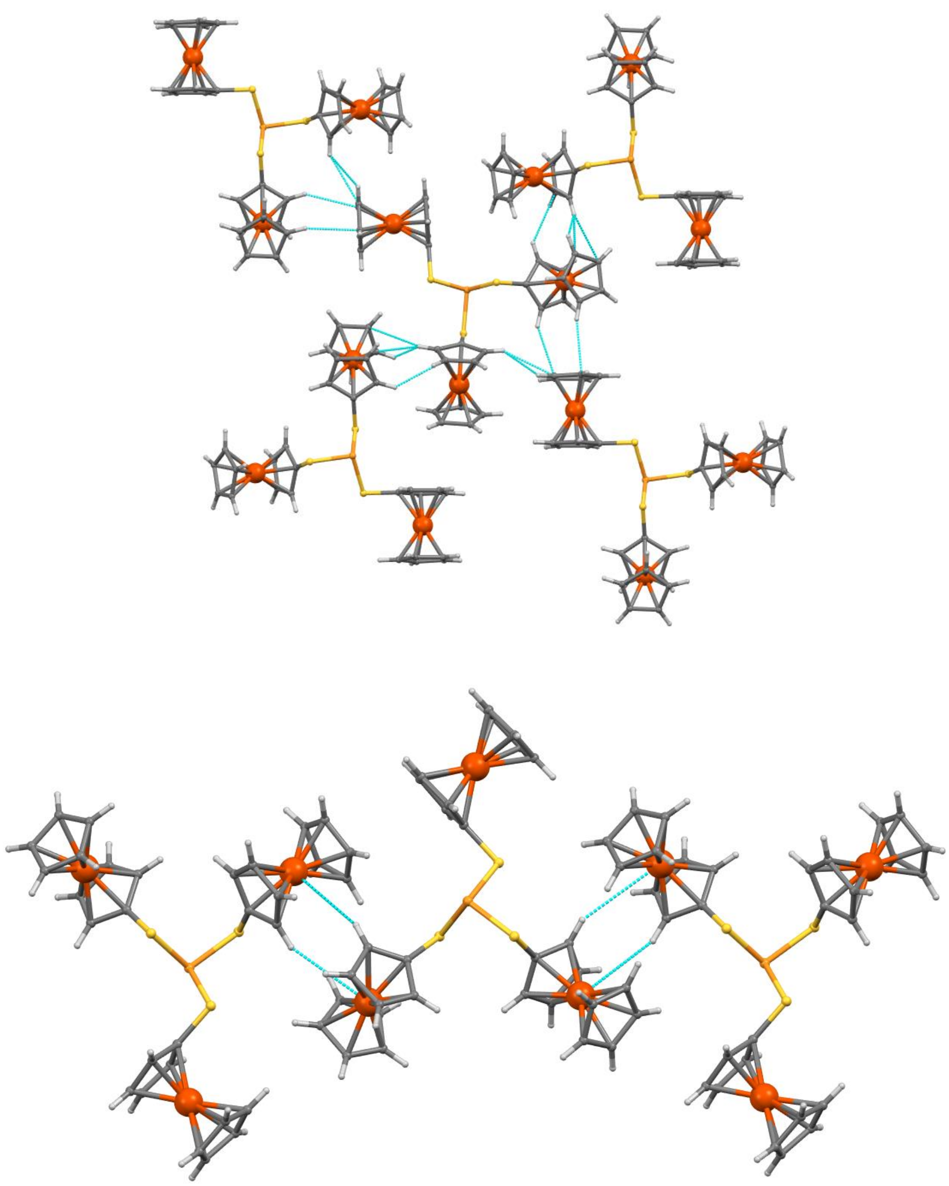

Figure S1: The crystal structure, packing and intermolecular interactions in crystal of $(FcS)_3P$.

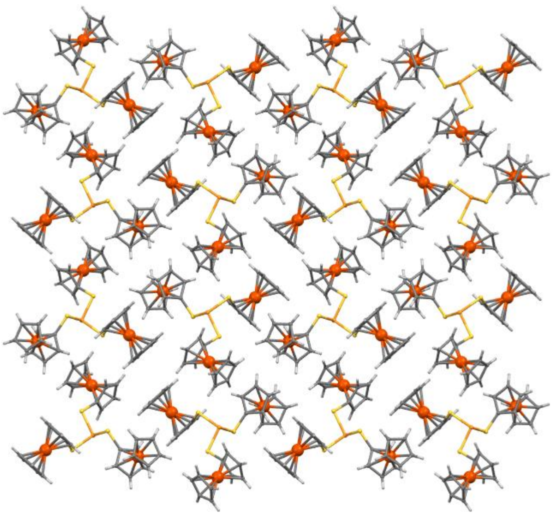

Figure S2: View along axis *a* in crystal of $(FcS)_3P$.

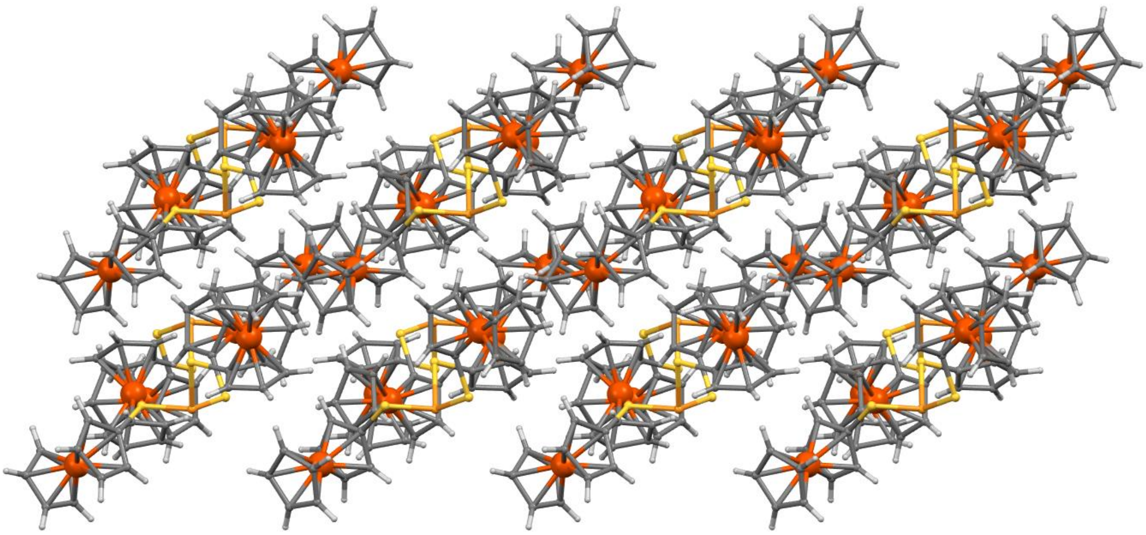

Figure S3: View along axis *b* in crystal of $(FcS)_3P$.

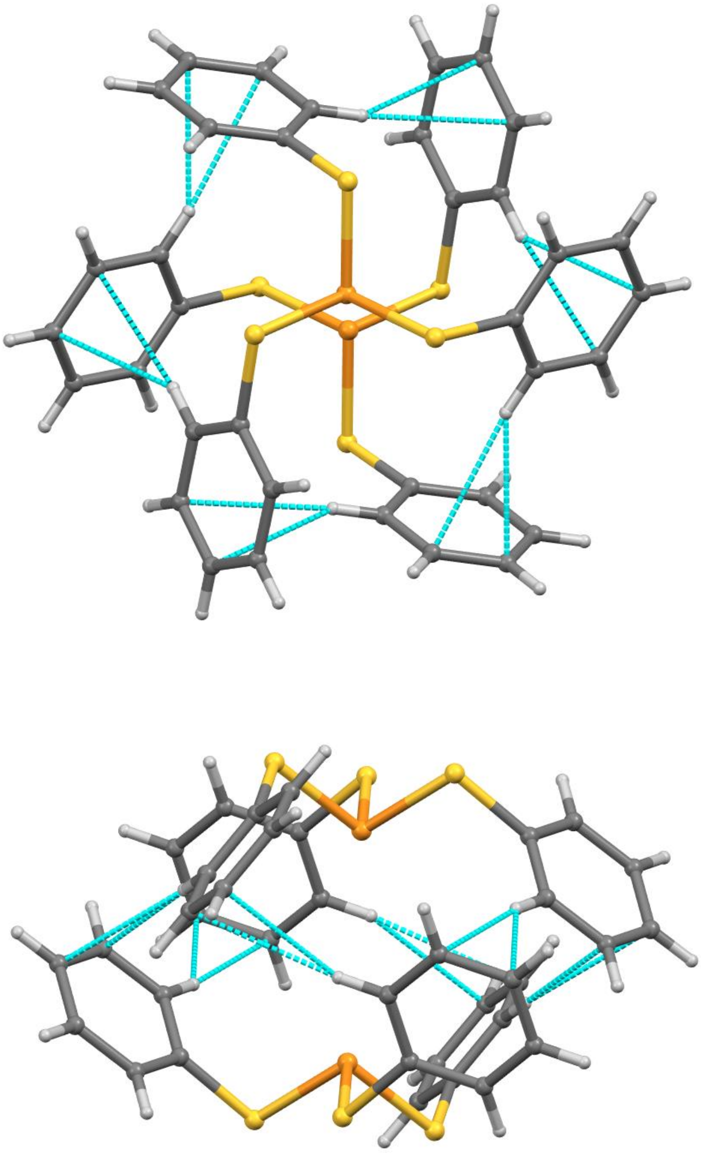

Figure S4: The crystal structure, packing and intermolecular interactions in crystal of $(PhS)_3P$.

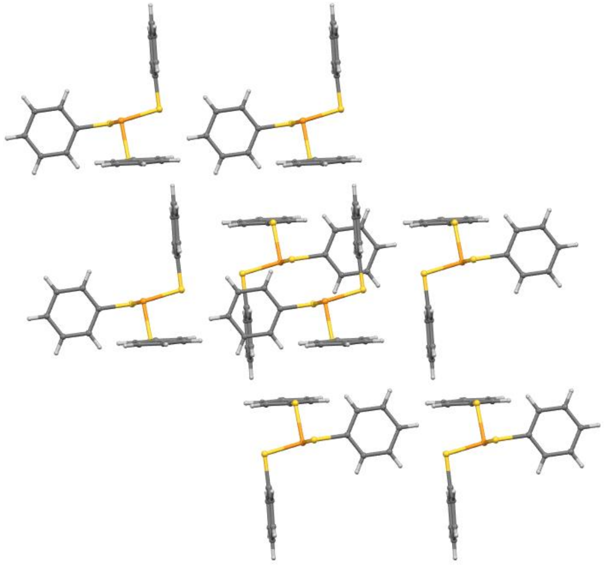

Figure S5: View along axis *a* in crystal of $(PhS)_3P$.
