# Supplementary material for: Comparison of crystal structure and DFT calculations of triferrocenyl trithiophosphite’s conformance
Source: Beilstein J Org Chem. 2022 Oct 25;18:1499–504. doi: 10.3762/bjoc.18.157 (PMC9643052; doi:10.3762/bjoc.18.157)
Supplement: File 3 — Check-CIF file for triferrocenyl trithiophosphite. [file Beilstein_J_Org_Chem-18-1499-s003.htm]

checkCIF/PLATON report


```
No syntax errors found.                               CIF dictionary  
Please wait while processing ....                     Interpreting this report
```

**Datablock: mil409\_corr**


---

|  |  |  |
| --- | --- | --- |
| Bond precision: | C-C = 0.0054 A | Wavelength=1.54184 |

|  |  |  |  |
| --- | --- | --- | --- |
| Cell: | a=7.4949(1) | b=19.8932(3) | c=18.4291(3) |
|  | alpha=90 | beta=99.792(2) | gamma=90 |
| Temperature: | 100 K |  |  |

|  |  |  |
| --- | --- | --- |
|  | Calculated | Reported |
| Volume | 2707.70(7) | 2707.70(7) |
| Space group | P 21/c | P 1 21/c 1 |
| Hall group | -P 2ybc | -P 2ybc |
| Moiety formula | C30 H27 Fe3 P S3 | C30 H27 Fe3 P S3 |
| Sum formula | C30 H27 Fe3 P S3 | C30 H27 Fe3 P S3 |
| Mr | 682.22 | 682.21 |
| Dx,g cm-3 | 1.673 | 1.674 |
| Z | 4 | 4 |
| Mu (mm-1) | 15.586 | 15.586 |
| F000 | 1392.0 | 1392.0 |
| F000' | 1386.35 |  |
| h,k,lmax | 9,25,23 | 9,24,23 |
| Nref | 5707 | 5496 |
| Tmin,Tmax | 0.124,0.292 | 0.194,0.609 |
| Tmin' | 0.019 |  |

|  |  |
| --- | --- |
| Correction method= # Reported T Limits: Tmin=0.194 Tmax=0.609 AbsCorr = GAUSSIAN |  |

|  |  |
| --- | --- |
| Data completeness= 0.963 | Theta(max)= 76.566 |

|  |  |
| --- | --- |
| R(reflections)= 0.0496( 4911) | wR2(reflections)= 0.1349( 5496) |
| |  |  | | --- | --- | | S = 1.062 | Npar= 334 | |

---

```
The following ALERTS were generated. Each ALERT has the format
       test-name_ALERT_alert-type_alert-level.
Click on the hyperlinks for more details of the test.


---

Alert level C
PLAT911_ALERT_3_C Missing FCF Refl Between Thmin & STh/L=    0.600          3 Report
PLAT977_ALERT_2_C Check Negative Difference Density on H9        .      -0.34 eA-3  


---

Alert level G
PLAT794_ALERT_5_G Tentative Bond Valency for Fe1       (II)      .       2.08 Info

And 2 other PLAT794 Alerts

PLAT794_ALERT_5_G Tentative Bond Valency for Fe2       (II)      .       2.11 Info  
PLAT794_ALERT_5_G Tentative Bond Valency for Fe3       (II)      .       2.10 Info

PLAT912_ALERT_4_G Missing # of FCF Reflections Above STh/L=  0.600        207 Note  
PLAT933_ALERT_2_G Number of HKL-OMIT Records in Embedded .res File          2 Note  
PLAT941_ALERT_3_G Average HKL Measurement Multiplicity ...........        3.1 Low   
PLAT978_ALERT_2_G Number C-C Bonds with Positive Residual Density.          0 Info  


---

   0 ALERT level A = Most likely a serious problem - resolve or explain
   0 ALERT level B = A potentially serious problem, consider carefully
   2 ALERT level C = Check. Ensure it is not caused by an omission or oversight
   7 ALERT level G = General information/check it is not something unexpected

   0 ALERT type 1 CIF construction/syntax error, inconsistent or missing data
   3 ALERT type 2 Indicator that the structure model may be wrong or deficient
   2 ALERT type 3 Indicator that the structure quality may be low
   1 ALERT type 4 Improvement, methodology, query or suggestion
   3 ALERT type 5 Informative message, check
```

---

---

It is advisable to attempt to resolve as many as possible of the alerts in all categories. Often the minor alerts point to easily fixed oversights, errors and omissions in your CIF or refinement strategy, so attention to these fine details can be worthwhile. In order to resolve some of the more serious problems it may be necessary to carry out additional measurements or structure refinements. However, the purpose of your study may justify the reported deviations and the more serious of these should normally be commented upon in the discussion or experimental section of a paper or in the "special\_details" fields of the CIF. checkCIF was carefully designed to identify outliers and unusual parameters, but every test has its limitations and alerts that are not important in a particular case may appear. Conversely, the absence of alerts does not guarantee there are no aspects of the results needing attention. It is up to the individual to critically assess their own results and, if necessary, seek expert advice. **Publication of your CIF in IUCr journals** A basic structural check has been run on your CIF. These basic checks will be run on all CIFs submitted for publication in IUCr journals (*Acta Crystallographica*, *Journal of Applied Crystallography*, *Journal of Synchrotron Radiation*); however, if you intend to submit to *Acta Crystallographica Section C* or *E* or *IUCrData*, you should make sure that full publication checks are run on the final version of your CIF prior to submission. **Publication of your CIF in other journals** Please refer to the *Notes for Authors* of the relevant journal for any special instructions relating to CIF submission. |

---

**PLATON version of 18/05/2022; check.def file version of 17/05/2022**

|  |
| --- |
| **Datablock mil409\_corr** - ellipsoid plot |
|  |

---

 Download CIF editor (publCIF) from the IUCr   
 Download CIF editor (enCIFer) from the CCDC   
 Test a new CIF entry 
